# A Rare Presentation of Myositis and Diffuse Alveolar Hemorrhage Associated With Disseminated Cryptococcus neoformans Infection

**DOI:** 10.7759/cureus.42062

**Published:** 2023-07-18

**Authors:** Srujan Edupuganti, Deepesh Yadav, Manoj Upadhyay, Andrew R Benck, Ailda Nika

**Affiliations:** 1 Internal Medicine/Pediatrics, Hurley Medical Center, Flint, USA; 2 Orthopedic Surgery, College of Medical Sciences, Kathmandu University, Bharatpur, NPL; 3 Internal Medicine, Hurley Medical Center, Michigan State University College of Human Medicine, Flint, USA; 4 Internal Medicine, Hurley Medical Center, Flint, USA; 5 Rheumatology, Rush University Medical Center, Chicago, USA

**Keywords:** diffuse alveolar hemorrhage, muscle biopsy, myositis, cryptococcus myositis, cryptococcosis, disseminated cryptococcus neoformans

## Abstract

Cryptococcosis is a fungal infection caused by species of the *Cryptococcus* genus which are commonly found in soil contaminated with bird feces, decaying wood, and tree hollows. It is usually seen in immunocompromised patients such as those with AIDS, with hematological malignancy, on immunosuppressive therapy, or after organ transplantation, and rare in immunocompetent hosts. The primary site of infection is usually the lung and the infection starts after inhalation of the pathogen and depending upon the host's immune response shows a different pattern of infection. Here we present a case report of a female in her late forties, who presented with two weeks of rash in her bilateral upper extremity, lower extremity, chest, and back along with arthralgia, myalgia, and proximal lower extremity weakness. Initial laboratory workup showed leukocytosis, elevated erythrocyte sedimentation rate, C-reactive protein, serum ferritin, and serum aldolase level with normal creatinine kinase. Rheumatological workups including ANA, ANCA, RF, C3, and C4 were normal. Magnetic resonance imaging of the right femur showed hyperintensity of the thigh and proximal calf musculature suggestive of muscle edema. A punch biopsy from the rash showed dyskeratosis with mild perivascular neutrophilic infiltrate. Steroid therapy and rituximab were started with some improvement. However, the patient developed respiratory distress and diffuse alveolar hemorrhage. Bronchoscopy was done and bronchoalveolar lavage fluid grew *Serratia* and *Candida*. The patient improved with antibiotic and antifungal therapy. However, the patient again developed respiratory distress and a new diffuse alveolar hemorrhage. A repeat bronchoscopy was done and the new bronchoalveolar lavage grew *Cryptococcus neoformans*. Blood cultures also grew *Cryptococcus neoformans*. The patient was started on amphotericin B and flucytosine. The patient initially improved and was transferred to the rehabilitation unit but ultimately her course was complicated by multiple infections and intubations and she unfortunately passed away.

## Introduction

Cryptococcosis is a potentially fatal invasive fungal infection caused by *Cryptococcus* species. Among all the cryptococcal species, *Cryptococcus neoformans* and *Cryptococcus gattii* are responsible for the majority of clinical illnesses in humans [1]. *Cryptococcus neoformans* are less likely to cause disease in immunocompetent people compared to *Cryptococcus gattii*. Disseminated *Cryptococcus* infection has been reported to affect immunocompetent hosts; however, it is more common in immunocompromised patients. The most common modes of transmission include exposure to soil contaminated with bird feces, decaying wood, and tree hollows [2]. Rarely, *Cryptococcus* infection can mimic the symptoms of dermatomyositis [3], which is the cornerstone of our case report.

## Case presentation

**Figure 1:**
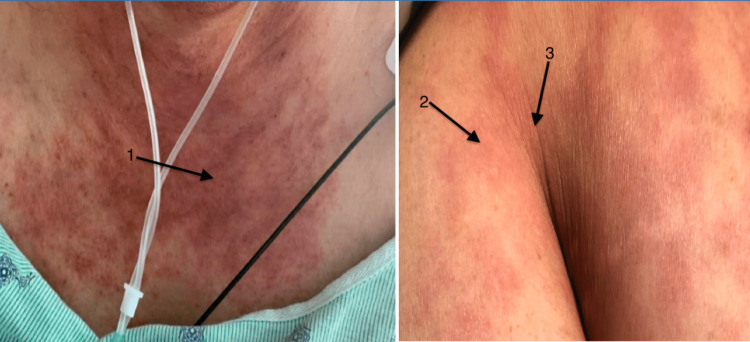
Rash seen on the chest, arm, and axilla as indicated by the arrows 1, 2, and 3, respectively

**Figure 2:**
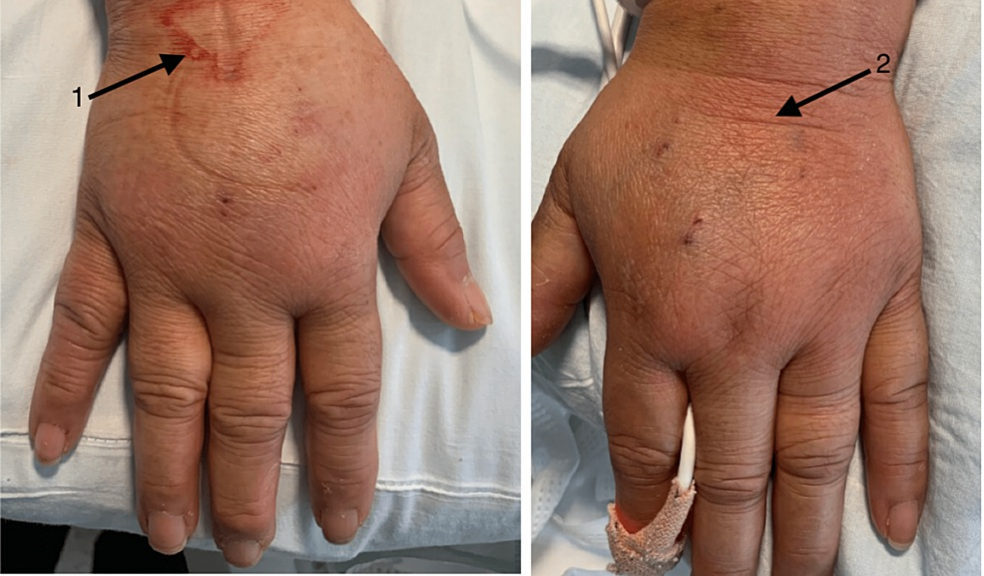
Rash over the right and left dorsal hands as indicated by the arrows 1 and 2, respectively

**Figure 3:**
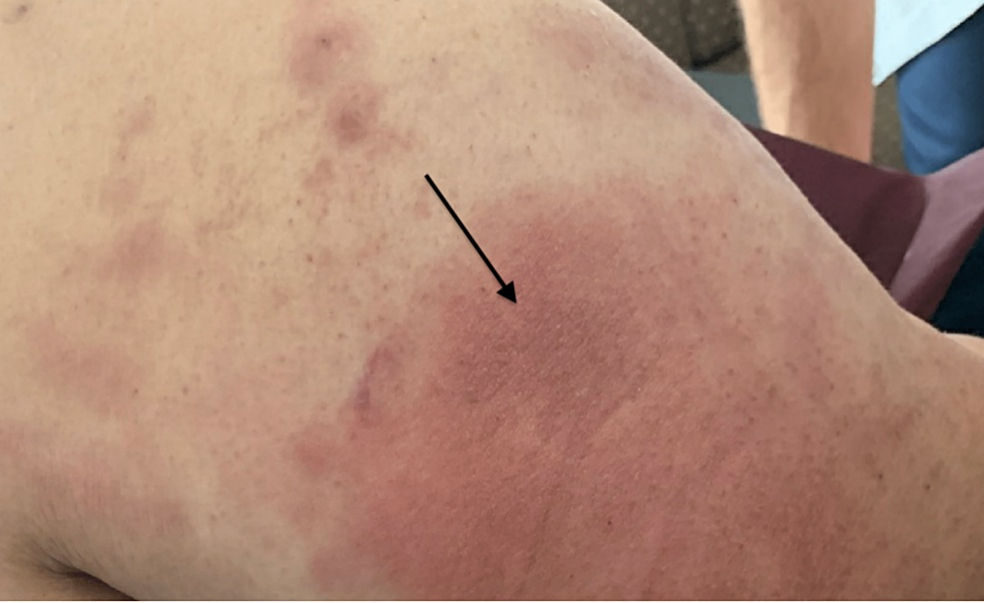
Back of the arm showing rash as indicated by the arrow

A female in her forties with a past medical history of hypertension, type 2 diabetes mellitus, hyperlipidemia, obesity, and asthma presented to the hospital with a two-week history of rash on her bilateral upper and lower extremities, chest, and back. In addition, she had arthralgia, myalgia, and proximal lower-extremity weakness. The initial exam was pertinent for the temperature of 103 degrees Fahrenheit, blood pressures ranging from systolic 86-136 and diastolic of 56-74 mmHg, tachycardia (with a heart rate range of 100-163 bpm), tachypnea (respiratory rate ranging from 18 to 48 per minute), and oxygen saturation between 92% and 98% in room air. Physical examination showed a salmon-colored blanching, nonpruritic, erythematous rash involving bilateral upper and lower extremities, chest, axillae, and back (Figures 1-3). The musculoskeletal examination was significant for swelling of the dorsum of the hands bilaterally without synovitis. All joints had a full range of motion both actively and passively and were without synovitis or effusions. Neurological examination showed 3+/5 motor strength in the proximal left hip flexors and 4/5 strength in the proximal right hip flexors.

Laboratory tests revealed leukocytosis with 24,600 white cells per microliter, erythrocyte sedimentation rate (ESR) of 106 millimeters per hour, C-reactive protein (CRP) 297 mg/L, and serum ferritin 18,000 micrograms per liter. Rheumatologic workup including rheumatoid factor, anti-neutrophil cytoplasmic antibody, antinuclear antibody, complement C3, C4, and creatinine kinase were normal. The thyroid-stimulating hormone was 0.22 micro IU/mL. Her initial presentation was concerning adult-onset Still's disease (AOSD) and she was started on prednisone 30 mg daily. However, the patient developed worsening transaminases with aspartate transaminase of 81 U/L, alanine transaminase of 52 U/L, and alkaline phosphatase of 163 U/L. The ferritin level increased from 18,000 mcg/L to 40,000 mcg/L. This worsening condition prompted the initiation of a pulsed dose of IV solumedrol 1 g/day for three days followed by prednisone 1 mg/kg/day and warranted further investigation. A punch biopsy of the rash was performed, which showed dyskeratosis with mild perivascular neutrophilic infiltrate. Computed tomography of the chest showed a large thyroid mass and enlarged mediastinal lymph nodes. Bone marrow biopsy was also done, which showed cellular marrow with trilineage maturation and flow cytometry negative for any kind of malignancy.

**Figure 4:**
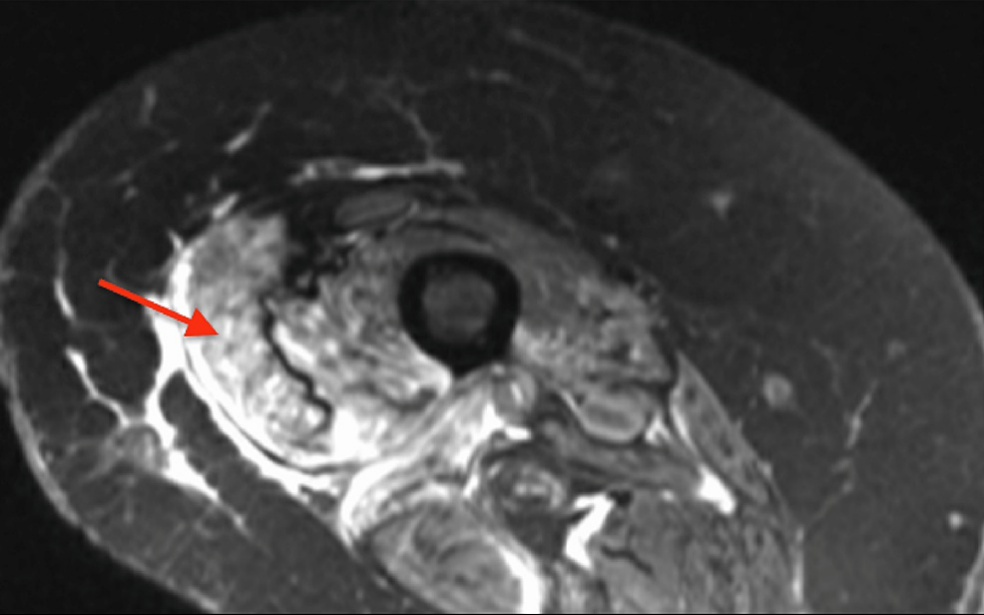
Magnetic resonance imaging with increased T2 signal intensity on the thigh musculature as indicated by the arrow

Hematology was consulted and determined that she did not meet the criteria for macrophage activation syndrome/hemophagocytic lymphohistiocytosis. With the initiation of high-dose steroids, her leukocytosis improved to 15k U/L, and serum ferritin trended down. However, she developed respiratory distress and bronchoscopy revealed diffuse alveolar hemorrhage (DAH). Plasma exchange was initiated. Her bronchoalveolar lavage fluid grew *Serratia* and *Candida*, which was not believed to be the cause of DAH by the Infectious Disease team. Her rash, lower-extremity proximal weakness, and diffuse alveolar hemorrhage raised concerns for dermatomyositis spectrum disease. Her creatinine kinase was within normal range but her aldolase was elevated to 30 units per liter. Magnetic resonance imaging (MRI) of the right femur showed hyperintensity of the thigh and proximal calf musculature suggestive of muscle edema (Figure 4). Based on these findings, a diagnosis of dermatomyositis was highly suspected and she was treated with rituximab, plasma exchange, and steroids. Following treatment, her skin rash resolved and her respiratory status improved to minimal oxygen requirements.

However, shortly prior to discharge, she again went into respiratory distress and a repeat bronchoscopy again showed DAH. Plasma exchange was started again and she remained on high-dose steroids. Because of her worsening condition, the diagnosis was questioned, and further workup was pursued. An EMG was negative for any irritative myopathy but a repeat MRI of the femur showed persistent muscle edema and aldolase remained persistently elevated to 21 units per liter. The repeat bronchoalveolar lavage grew *Cryptococcus neoformans* and blood cultures also grew *Cryptococcus neoformans*. She was started on amphotericin B and flucytosine for disseminated cryptococcal infection. The patient initially improved but ultimately her course was complicated by multiple infections and intubations and she unfortunately passed away.

## Discussion

Although rare, *Cryptococcus* has been associated with infections in immunocompetent hosts. A retrospective case series was performed at two hospitals in Hong Kong from 1995 to 2005, which included 46 patients with *Cryptococcus*. Inclusion criteria were clinical and/or radiological features indicative of acute cryptococcal infection and the infection was confirmed by culture/histology. Forty-three percent of the patients were immunocompetent and 57% were immunocompromised (37% had one or more predisposing factors other than HIV and 20% of the patient had HIV). Cryptococcal infection can present as pneumonia or with various neurological manifestations, including meningitis, myelitis, encephalitis, and cryptococcoma [4]. The most common presentation in immunocompetent patients was meningitis (80% compared to 57% in immunocompromised patients) and extrapulmonary manifestations (47%). Ten percent of the patients presented with pulmonary *Cryptococcus*, and 10% of the immunocompetent patients presented with extraneural and extrapulmonary manifestation. Immunocompetent patients presented with lower rates of fungemia compared to immunocompromised (10% vs 35%) [5].

Myositis due to infectious causes has been reported in a few patients. Cryptococcal myositis is an extremely rare condition, and it has been reported only a few times before the autopsy of patients after death. Most of these patients had a negative outcome with death secondary to comorbidities like cardiac arrest rather than the disease process or inadequate response to the therapy [6,7]. It is a rapidly worsening condition and requires immediate attention and management. Most of the patients start with asymmetric, localized muscle swelling associated with muscle pain. The most common muscle groups are buttocks and thigh calf in 80-90% of patients [8]. Some patients might have minor trauma, to begin with. Patients may present with fever, elevated WBC, and elevated markers of inflammation (ESR, CRP). Muscle enzyme level is usually normal at the time of presentation. Histologic evaluation of tissue is an alternative method of detecting fungal organisms and remains a strong adjunct to microbiologic culture for diagnosis. It is usually visualized on hematoxylin & eosin (H&E) stains and then confirmed with direct visualization via specific stains for fungus, commonly GMS (Grocott’s methenamine silver) or PAS (periodic acid-Schiff), in tissue and cytology specimens [9]. The initial modality of testing is MRI with a T1-weighted image showing increased muscle size and T2 images showing increased signal intensity. However, muscle biopsy is helpful to determine the specific pathogen [10-12]. However, the results can be equivocal in patients with steroid treatment. We treated the patient with steroids and that itself might have contributed to the inconclusive muscle biopsy results and delay in the initiation of treatment.

A case report was published in 2016 of a 44-year-old gentleman with AOSD who presented with fever, dry cough, and dyspnea of two weeks duration. Methotrexate and prednisone were started. His respiratory status worsened and the CT chest showed bilateral patchy infiltration and ground-glass appearance consistent with the radiological findings of acute respiratory distress syndrome. He was intubated and underwent an open lung biopsy and the pathology showed diffuse, interstitial inflammation along with fibroblastic proliferation in the lung parenchyma, hyperplasia of the alveoli epithelium, and some remnants of hyaline membranes as well. Histopathological findings were compatible with diffuse alveolar damage and the cultures grew *Cryptococcus* [13].

DAH is associated with a number of clinical entities and several histological subtypes. The most common underlying histological finding is small-vessel vasculitis and DAH due to a number of injuries including drugs, coagulation disorders, and infections. A case series was published in 2022, which involved 58 patients with DAH. The patients underwent surgical lung biopsy over a seven-year period (1996-2002) to identify the cause. CT chest findings showed predominantly ground-glass and consolidative opacity. The study showed that the most common causes of DAH in these patients were infectious conditions (22%) similar to this patient, noninfectious pulmonary complications, transplant (17%), connective tissue disease (16%), acute exacerbation of idiopathic pulmonary fibrosis (12%), acute interstitial pneumonia (with no identifiable cause or predisposing condition for DAH, 12%), drugs (10%), and radiation therapy (2%) [14].

The drug of choice for initial therapy in disseminated cryptococcosis is amphotericin B with flucytosine. The main role of flucytosine is to decrease the rate of treatment failure and increase the survival rate. Fluconazole has very good cerebrospinal fluid penetration; however, since it is only fungi static it is not the choice during the induction phase, but during the consolidative and suppressive phases, it is very useful in combination with amphotericin B [5].

## Conclusions

Due to this unusual presentation and culture eventually growing *C. neoformans,* this case is very unique in its own way. Disseminated *Cryptococcus infection* with manifestations similar to our patients are very sparse in the literature and any patient who presents with muscle weakness, fever, and DAH with elevated inflammatory markers (ESR, CRP, ferritin) along with raised aldolase level should be investigated for infectious as well as inflammatory cause from the beginning and treatment should be planned accordingly.

## References

[1] Chang CY, Mohd Shah SH, Lio JY, Bahari N, Radhakrishnan AP (2021). Cryptococcus gattii meningitis complicated by immune reconstitution inflammatory syndrome in an apparent immunocompetent host in Malaysia. Med Mycol Case Rep.

[2] Mada PK, Jamil RT, Alam MU (2022). Cryptococcus. StatPearls [Internet].

[3] Lortholary O, Nunez H, Brauner MW, Dromer F (2004). Pulmonary cryptococcosis. Semin Respir Crit Care Med.

[4] Chang CY (2021). Cryptococcal meningoradiculitis presenting with acute flaccid paralysis. Rev Soc Bras Med Trop.

[5] Lui G, Lee N, Ip M (2006). Cryptococcosis in apparently immunocompetent patients. QJM.

[6] Lewis JL, Rabinovich S (1972). The wide spectrum of cryptococcal infections. Am J Med.

[7] Wrzolek MA, Sher JH, Kozlowski PB, Rao C (1990). Skeletal muscle pathology in AIDS: an autopsy study. Muscle Nerve.

[8] Flagg SD, Chang YJ, Masuell CP, Natarajan S, Hermann G, Mendelson MH (2001). Myositis resulting from disseminated cryptococcosis in a patient with hepatitis C cirrhosis. Clin Infect Dis.

[9] Haque AK, McGinnis MR (2008). Fungal Infections. Dail and Hammar’s Pulmonary Pathology.

[10] Gomez-Reino JJ, Aznar JJ, Pablos JL, Diaz-Gonzalez F, Laffon A (1994). Nontropical pyomyositis in adults. Semin Arthritis Rheum.

[11] Gibson RK, Rosenthal SJ, Lukert BP (1984). Pyomyositis. Increasing recognition in temperate climates. Am J Med.

[12] Beltran J (1995). MR imaging of soft-tissue infection. Magn Reson Imaging Clin N Am.

[13] Orsini J, Blaak C, Tam E, Rajayer S, Morante J (2016). Disseminated cryptococcal infection resulting in acute respiratory distress syndrome (ARDS) as the initial clinical presentation of AIDS. Intern Med.

[14] Parambil JG, Myers JL, Aubry MC, Ryu JH (2007). Causes and prognosis of diffuse alveolar damage diagnosed on surgical lung biopsy. Chest.

